# From research to policy: enhancing uptake of quality improvement methods in government contracts

**DOI:** 10.1186/1748-5908-8-S1-S8

**Published:** 2013-04-19

**Authors:** Heather L Tubbs-Cooley, Joanne Lynn

**Affiliations:** 1 Department of Patient Services & the James M. Anderson Center for Health Systems Excellence, Cincinnati Children's Hospital Medical Center, Cincinnati, Ohio, 45229, USA; 2 Center for Elder Care and Advanced Illness, Altarum Institute, Washington, DC, 20036, USA

## Presentation

The Centers for Medicare and Medicaid Services (CMS) provide healthcare coverage for 100 million people and, particularly through provisions of the Affordable Care Act, the agency strives to improve care and to ensure coverage for all Americans. Government agencies like CMS need processes that encourage improvements in value and outcomes and reduce variation in quality, and yet have been slow to embrace quality improvement (QI) methods. With QI, the agency could be more effective in partnering with providers to achieve “triple aim” outcomes of improving patient experiences with health care, improving population health, and reducing per-capita health care costs [1].

Most CMS work proceeds through contracts that specify actions and on-time deliverables (such as supplies or helpdesk services). Similarly, contracts to “Quality Improvement Organizations” (QIOs) typically require that an “evidence-based” intervention be applied in a certain number of clinical settings – not that the intervention be tested further and adapted to the local context, or even that a particular outcome be achieved. Such contracts are eminently auditable, an important fact in the scrutiny of government contracting by CMS, Congress, the press, and others.

Translating efficacious interventions into effective health care processes and outcomes at a local level ordinarily requires iterative, exploratory testing and adaptation, which is the core of QI. CMS’s traditional purpose has been to pay the bills and uphold the “standard of care”; it generally does not issue research grants that allow exploration of novel implementation approaches. Although CMS has not historically been at the forefront of QI methods, the agency’s position is simultaneously changing to adopt QI and encountering challenges along the way.

Key points:

• Implementation of evidence-based interventions with strict fidelity to the research protocol is often an ineffective strategy; testing and adaptation are usually necessary for optimal implementation and for scaling up.

• Quality improvement methods such as statistical process control (SPC), frequent and repeated measurement, rapid-cycle testing of interventions and strategies, and qualitative insights about causal chains and effectiveness are powerful implementation tools that could work better to achieve program goals than implementation of rigidly specified interventions.

• Writing an auditable contract for these approaches poses challenges.

• Strategic partnerships between QI researchers, QI leaders, and government staffers/officials might be effective in promoting familiarity with QI methods and structuring contracts to allow for integration of QI methods while meeting audit and evaluation needs.

## Commentary

A contemporary example of a large-scale CMS program that presents learning opportunities related to effective implementation of interventions is the work on care transitions, culminating in the current Community-based Care Transitions Program (CCTP). Created by the Affordable Care Act, CCTP is a $500 million program designed to engage hospitals and community-based organizations to improve care transitions from the hospital to other settings and thereby to reduce readmissions for high-risk Medicare beneficiaries. In 2006 as part of the work leading to the CCTP, CMS devised tests of two concepts: 1) Would practitioners and health systems be willing to work with CMS on projects related to value? and 2) Would the proposed evidence-based care transition interventions be effective in reducing hospital readmissions? The answer to both questions was yes; 20 hospitals participated and, within six months, several hospitals demonstrated a 30-50% reduction in readmission rates. The pilot tests encouraged the use of QI methods and created the opportunity to work in geographic communities rather than with targeted providers alone.

Thus, through the 9^th^ Scope of Work (SOW), 2008-2011, 14 QIOs were selected to participate in a novel endeavor to improve care transitions in geographic communities [2]. That project required QI method landmarks to achieve particular levels by 18 months into the contract. For example, QIOs were required to report monthly the proportion of transitions of fee-for-service Medicare patients that were (1) represented by the providers participating in the improvement, (2) affected by an implemented intervention, and (3) measured for effect, and to achieve rates above 30%, 25%, and 10% respectively. Through this enforced QI approach, all sites exceeded these rates. By 28 months, rates of 30-day readmissions were required to decline by at least 2%. The QIOs had a menu of evidence-based interventions to use [3], as well as QI practices such as process mapping and process standardization, but they were free to engage fully with their clinical partners in figuring out what would motivate the will to change and how to adapt, test, and monitor daily work.

The project succeeded in reducing hospitalization and re-hospitalization and this led to the CCTP, which appears likely to fund approximately 102 programs. Indeed, CMS proposes a massive scale-up from 14 communities to national implementation. However, the current projects have retreated somewhat from the QI methods that were intrinsic to the 9^th^ SOW. The current contracted projects have specific deliverables and timetables and the CCTP is structured as a CMS payment plan. Technical support and evaluation contracts include language about learning organizations, but the contract and payment structures do not appear to support substantial testing, innovation, close monitoring, or rapid learning, and no central authority nationally or at the state level is situated to shape the testing of hunches and hypotheses [4].

## Recommendations

Integration of QI methods into the federal government’s contracting tool kit would allow CMS and other agencies to build insight from natural learning opportunities within projects that are conducted in complex settings and diverse populations and communities. To facilitate uptake of these methods, we make the following recommendations:

• The Academy for Healthcare Improvement and CMS should work together to develop, discover, and catalog useful strategies for contracting that can encourage QI, e.g., cooperative agreements with many checkpoints, process measures, timely SPC charts, and reports of local insights.

• Insights from agencies already using some QI methods (e.g., the Veterans Health System, the Indian Health System, and the Agency for Healthcare Research and Quality) should be sought, documented, and disseminated.

• As these initiatives develop, project officers and contracting staff, as well as staff in oversight agencies and Congress, will need education about the processes and their merits.
